
JOURNAL INFORMATION
==============================
NLM Title Abbreviation: Behav Neurol
Journal ID: Behav Neurol
NLM Journal ID: 8914585
Journal ID: BN

Behavioural Neurology

ISSN: 0953-4180
EISSN: 1875-8584
Publisher: Wiley

ARTICLE INFORMATION
==============================
PMCID: PMC5507120
PMID: 12440471
DOI: 10.1155/2002/407056
Article ID: 407056
Article version: 1
Subjects: Editorial

Motor Aspects of Hemispatial Neglect

Jackson Stephen R.
School of Psychology The University of Nottingham University Park Nottingham NG7 2RD UK
Print publication date: 2002
Electronic publication date: 2002 Oct 1
Volume: 13
Issue: 1-2
First page: 1
Last page: 2
Received 2002 Jul 2; Accepted 2002 Jul 2
Copyright: Copyright © 2002 Hindawi Publishing Corporation.
Copyright year: 2002
License: This is an open access article distributed under the Creative Commons Attribution License, which permits unrestricted use, distribution, and reproduction in any medium, provided the original work is properly cited.
License URL: https://creativecommons.org/licenses/by/3.0/

==============================
Behavioural Neurology 13 (2001/2002) 1–2
IOS Press

Editorial

### Motor aspects of hemispatial neglect

Stephen R. Jackson
*School of Psychology, The University of Nottingham, University Park, Nottingham NG7 2RD, UK*
*E-mail: Stephen.Jackson@nottingham.ac.uk*

A relatively common outcome of unilateral brain damage in humans is the syndrome of hemispatial neglect. Hemispatial neglect can occur following damage to a variety of brain regions but is chiefly associated with damage to the inferior parietal lobule, most frequently involving the occipito-temporo-parietal junction of the right hemisphere [1]. Neglect patients fail to respond appropriately to stimuli or events occurring within their contralesional hemispace, and may restrict eye and hand movements to objects or events located within ipsilesional space. Such behaviours are generally thought to result from an impairment in the ability to construct an appropriate representation of corporeal and extrapersonal space, or, as a consequence of an attentional bias which favours the processing of ipsilesional stimuli. However, neglect patients may also suffer from a disorder of 'intention', experiencing difficulty initiating movements towards targets presented within their neglected hemispace. It is the nature of any motor impairments associated with hemispatial neglect that is the focus of this special issue of Behavioural Neurology.

While it is generally accepted that the syndrome of hemispatial neglect may reflect several underlying impairments, there has been a longstanding and influential view that a substantial number of neglect patients present with some form of motor bias. Right hemisphere patients presenting with left neglect have been shown to be slow in initiating leftward compared to rightward movements [e.g., 2,3], however, such studies have proven difficult to interpret as the movements in these studies are invariably cued by visual targets. Thus, it remains highly plausible that much of the motor impairment observed in hemispatial neglect has a perceptual basis, resulting in an impairment in the representation of space used to guide action [4].

If it is indeed the case that some aspects of hemispatial neglect may be attributable to a distorted representation of corporeal and extrapersonal space, then a critical question is whether such distorted perceptions lead to erroneous visuomotor behaviour. According to Milner and Goodale's influential 'two visual systems' account [5], such perceptual distortions should affect perceptual judgments (e.g., explicit judgments of object size), but would be expected to have minimal effect on visuomotor actions directed to these same objects. This issue is explored further in the papers by McIntosh et al., Harvey et al., and Gore et al. which each examine the effects of egocentric location on the kinematics of reach-to-grasp movements and report the relative sparing of visuomotor performance.

A related aspect of motor bias in hemispatial neglect concerns the paucity of leftward movements exhibited by neglect patients during spatial search tasks [6,7]. Thus, during both visual [6] and tactile [7] search tasks, the centres of exploration of space are shifted towards the right in neglect patients for both exploratory eye and hand movements. This issue is re-examined in the papers by Behrmann et al., and di Pelligrino et al.

Behrmann et al., investigate whether the paucity shown by neglect patients in executing leftward saccades during spatial search tasks reflects a deficit in perception, in saccade planning, or in saccade execution. They report that patients with left neglect are impaired in planning but not in executing the contralesional saccades, and suggest that parietal cortex may play a specific role in directing action to different sides of space. The topic of ocular exploration of space is

ISSN 0953-4180/01/02/$8.00 © 2001/2002 – IOS Press. All rights reserved also addressed in the paper of di Pelligrino et al., who investigate the effects of hemispatial neglect on the allocation of spatial attention over letter strings during reading. di Pelligrino et al. report a single case study of a patient (F.C.) who presents with a severe left neglect dyslexia who was required to read isolated word and non-word stimuli. They report that their patient's ocular exploration of orthographic stimuli is sensitive to the lexical status of the letter string. Specifically, their patient spends more time fixating the contralesional side of word than non-word strings. They also demonstrate that patient F.C. shows a dissociation between conscious access to orthographic stimuli and his eye movement responses.

di Pelligrino et al. propose that multiple interactions between lexico-semantic, attentional and motor systems may influence the presentation of neglect during search tasks. This suggestion finds additional support in the case study reported by Humphreys and Riddoch. These authors examined visual search in a patient (MP) presenting with left hemineglect. They varied how target items were defined – i.e., whether a target was defined by a description of its action or its name – along with the number of targets and distractors presented, and whether search was for multiple or single targets. Humphreys and Riddoch report that search was substantially improved when targets were defined by a description of their action rather than their name. The authors propose that search may be based on action-defined templates of targets, which may be activated by the action affordances.

The collection of papers presented within this special issue is not intended to be an exhaustive overview of the range of deficits associated with 'motor' aspects of hemispatial neglect. Instead the collection provides illustrative examples of current research within this rapidly changing field of enquiry.

### References

[1] G. Vallar, Spatial hemineglect in humans, *Trends in Cognitive Sciences* **2** (1998), 87–97.

[2] K.M. Heilman, D. Bowers, H.B. Coslett, H. Whelan and R.T. Watson, Directional hypokinesia: prolonged reaction times for leftward movements in patients with right hemisphere lesions and neglect, *Neurology* **35** (1985), 855–859.

[3] J.B. Mattingley, J.L. Bradshaw and J.G. Philips, Impairments of movement initiation and execution in unilateral neglect, *Brain* **115** (1992), 1849–1874.

[4] S.R. Jackson, R. Newport, M. Husain, M. Harvey and J.V. Hindle, Reaching movements may reveal the distorted topography of spatial representations after neglect, *Neuropsychologia* **38** (2000), 500–507.

[5] A.D. Milner and M.A. Goodale, *The visual brain in action*, Oxford: Oxford University Press, 1995.

[6] H.-O. Karnath, M. Niemeier and J. Dichgans, Space exploration in neglect, *Brain* **121** (1998), 2357–2367.

[7] H.-O. Karnath and M.-T. Perenin, Tactile exploration of peripersonal space in patients with neglect, *NeuroReport* **9** (1998), 2273–2277.
